# George Voronovsky: maker of memoryscapes

**DOI:** 10.1017/S2045796023000288

**Published:** 2023-06-06

**Authors:** Katherine Jentleson, Kyle Mancuso

**Affiliations:** Folk and Self-Taught Art, High Museum of Art, Atlanta, GA, USA

**Figure 1.**
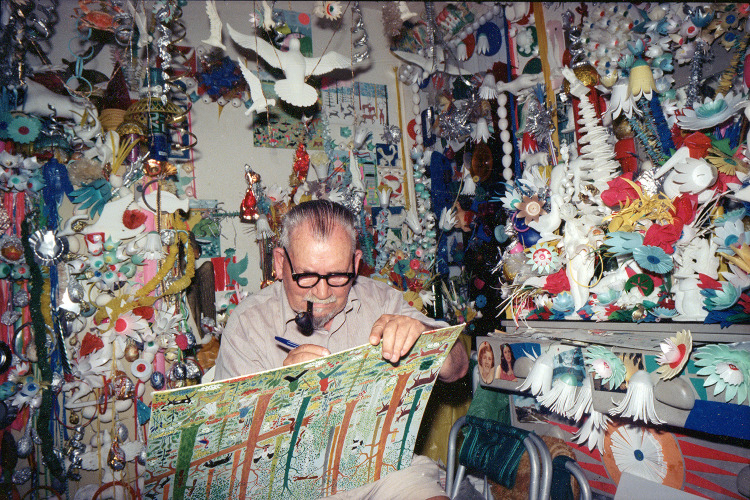
Portrait of George Voronovsky, 1978–1982, by Gary Monroe (American, born 1951).

In 1972, Ukrainian-born self-taught artist George Voronovsky permanently moved from Philadelphia to Miami, taking up long-term residence in a room on the third floor of the Colony Hotel with a window that overlooked a beautiful stretch of South Beach. Over the next decade, he filled his small space with hundreds of artworks, covering his walls with dense mosaics of paintings and hand-carved Styrofoam sculptures (Figure 1). Rainbow flowers cut from tin cans burst from the layered surfaces of the walls while coffee tin lids and discarded plastic bottles swung in front of the windows, constructing a space that one journalist of the period aptly described as a ‘defiant oasis of color and light’ (McLeod, 1987).

When Voronovsky was about 6 years into transforming this space, Gary Monroe, a Florida-based photographer who grew up blocks from the Colony Hotel, spied Voronovsky’s decorated room from the sidewalk. ‘There were bright, cheerful-looking objects dangling in the open windows there. They seemed to march off into its darker recesses in a whimsical procession,’ he recalled several years later. ‘It looked as if a rainbow had been broken into fragments and hung out to dry’ (McLeod, 1987). Together with a circle of similarly creative companions, Monroe befriended Voronovsky and ultimately became the champion of his legacy, storing his art and personal archive for over 40 years. His work was shown only a handful of times, and on a small scale, since his death in 1982. In March of 2023, the High Museum of Art opened the first major solo exhibition on Voronovsky’s artwork, presenting his *memoryscapes* of a happily remembered, if tumultuous, life in Ukraine at a time when that nation is once again faced with the destruction of its people, landscapes and cultural identity.

Voronovsky’s Colony Hotel room – transformed into an ‘oasis’ – is an example of an artist-built environment, an ever-changing space comprising many singular works of art whose aggregate often only survives so long as the artist does. Voronovsky’s environment – constructed in the Colony, then reconstituted in a senior living facility in his final years, and finally dismantled upon his death – was created as a place of respite from the traumas of his past: revolutions, famines, world wars and forced migrations among them. Monroe attests that Voronovsky abhorred the whitewashed walls of the Colony, which may have reminded him of the drab and stifling environments of the Nazi resettlement and labour camps he experienced during the Second World War (Lacher, 1986). Surrounding himself with an arrangement of joyful scenes brimming with colour was the ultimate antidote for a life whose trajectory was disrupted by conflict and displacement.

Voronovsky was born in Klepaly, a small village in eastern Ukraine in what was then part of the vast territories of the Russian Empire. Around the age of 14, Voronovsky lost his father during the upheavals of the First World War and the Soviet–Ukraine War. When he was 38 years old, Nazi forces invaded Kyiv, forcing him to leave Ukraine for a series of resettlement and labour camps in occupied Europe. Sceptical of the Soviet regime that controlled much of Eastern Europe after the war – and that considered many forced labourers not as victims but as traitors – Voronovsky refused repatriation to Ukraine and lived between Displaced Persons camps in Germany while awaiting resettlement, by this time separated from his family. In 1951, with the help of international relief agencies and American humanitarian organizations, Voronovsky was able to immigrate to the United States, passing through Ellis Island and eventually settling in Philadelphia. There, living in one of the largest Ukrainian communities in North America, he had the opportunity to spend time with other recent immigrants and would have been exposed to folk festivals and art shows supported by the area’s churches and cultural institutions (Pearse, 1956). For the next 20 years, Voronovsky worked in the railroad industry cleaning and upholstering train cars. He also found time to explore sites up and down the East Coast, taking the Greyhound bus to destinations as far apart as Niagara Falls and Florida and capturing these trips on a 16 mm film and in photographs. (Though many questions remain about Voronovsky’s past, what is known about his life can be explored in greater detail through an interactive timeline on the High Museum of Art’s website.)

Some of Voronovsky’s earliest surviving work dates to this period. Wood-carved figures of nude bathing women that hearken back to the American folk-art traditions of the Pennsylvania Dutch filled is Philadelphia home. An experimental film about a trip to the sun aboard a spaceship, starring Voronovsky, was shot in the woods behind the company rail yard. The bulk of his artwork, however, dates to his years in Miami Beach and recalls the colours and motifs of Ukrainian folk-art.

In the heart of the South Beach neighbourhood – a seaside resort populated by Eastern European immigrants of his generation, many of them Jewish, others Orthodox Christian like himself – Voronovsky embraced the artistic potential of the materials that surrounded him. In one of the rare documents that he penned in English – he wrote mostly in Russian – he describes how he embraced discarded things: ‘I like to do decoration for my room from […] what is throwen [*sic*] away’. Nearly all of the paintings that he would create over the next decade depicted the landscapes and cultural traditions of his beloved Ukraine, but in an exercise of double consciousness that is typical of the immigrant experience, he created art that embodied both his Ukrainian past and American present. He painted a Ukrainian idyll, for example, on a grease-stained Pizza Hut takeout box and carved birds of Slavic mythology out of discarded Styrofoam coolers, salvaged during his daily walks on the beach. Aluminum cans that once held Budweiser and Sunkist were cut into star shapes that recall the folk-infused fireworks displays in his painting (Figure 4).

His reuse of packaging related to the abundance of consumables that surrounded him in South Beach allowed him to transform his room into ‘an enchanted forest’ fashioned from ‘the discards of fast-food America’ (McLeod, 1987). Voronovsky did not speak openly about his young adulthood living in Soviet-controlled Ukraine, including how he must have experienced the Holodomor, the forced starvation engineered by the Soviet regime that killed millions of Ukrainians in 1932–33 (Applebaum, 2017), as well as food scarcity during subsequent Nazi internment. With this past suffering in mind, the excess of fatty foods and sugary drinks Voronovsky encountered in the booming post-war capitalism of the United States, and his decision to transform its containers into art, takes on a new dimension.

**Figure 2.**
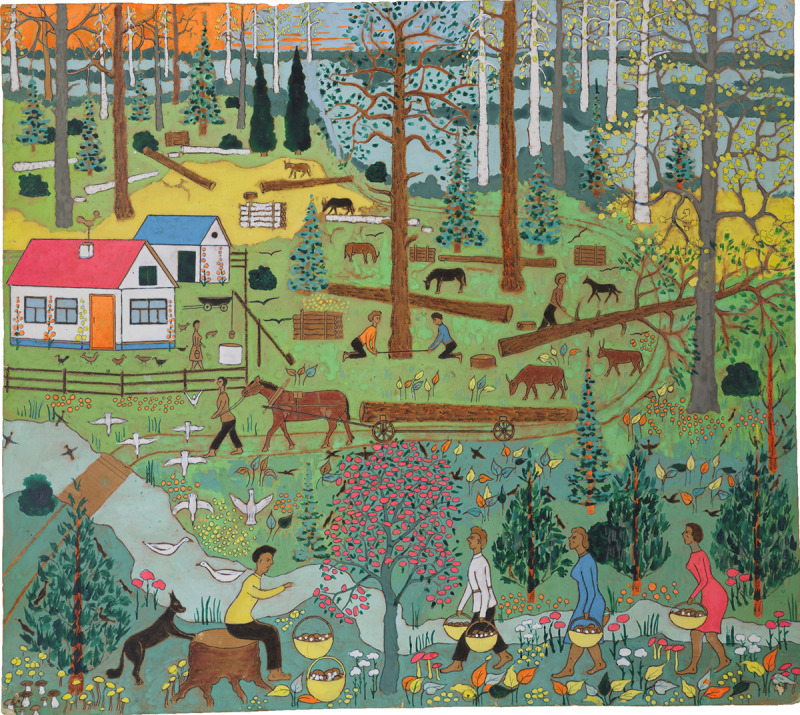
*Untitled (Gathering Mushrooms)*, 1972–1982, paint on cardboard, courtesy of the Monroe Family Collection.

Such creative scavenging is not of course limited to Voronovsky, who belongs to a tradition of self-taught artists who found in discarded materials both economy and a challenge. But Voronovsky – who grew up in a middle-class family that spent time in both metropole and periphery – had been scavenging all his life, as familiar folk-art traditions of Ukraine like egg painting (*pysanky*), allusions to which abound in his work, required eggs to paint on. References to these foraging excursions show up throughout his art. In one painting, Voronovsky and his brothers march through a densely wooded forest in search of eggshells and snakeskins ‘to have the beauty of nature’, as he inscribed on the verso. In another, a typical Eastern European countryside custom – gathering mushrooms – is painted on the cardboard packaging of a plastic floatie (Figure 2). With no intention of selling his paintings or storing them away, Voronovsky pinned these makeshift canvases to his whitewashed walls with thumbtack – the holes made by these punctures are still visible on many of his cardboard paintings – creating dialogues between disparate places and memories.

**Figure 3.**
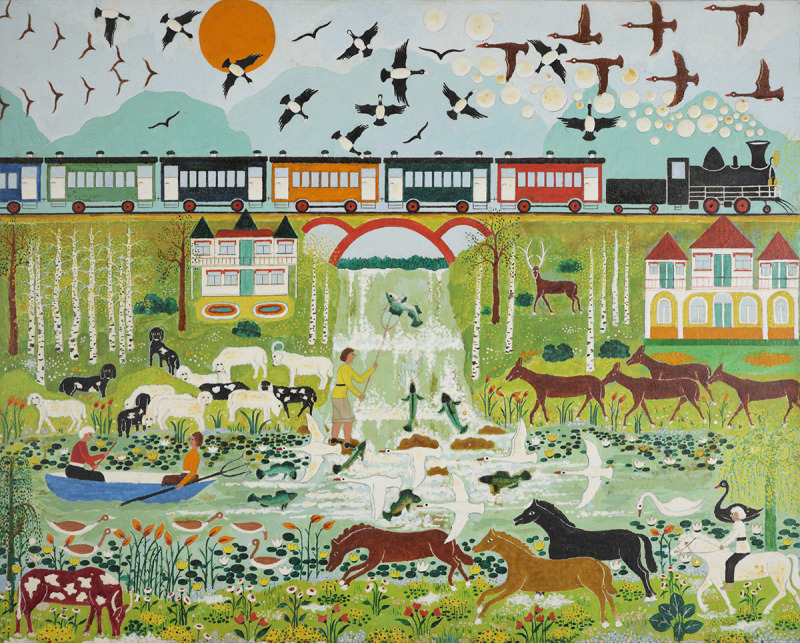
*Untitled (Trout Fisherman)*, 1978–1982, acrylic on canvas, courtesy of the Monroe Family Collection.

Scavenged materials had their limitations, however. Watered-down paint from the local five-and-dime has a tendency to bleed, and cardboard is wont to curl up. It was not until the late 1970s, with the help of Monroe and friends, that Voronovsky applied for and received a grant from the Florida Arts Council, giving him the financial means to purchase higher-quality materials like acrylic paints and pre-stretched canvas. With these sturdier paints, Voronovsky concocted more polished scenes and added rich textures to his paintings, creating a sense of depth out of the froth of a waterfall and the steam bubbles of a passing locomotive (Figure 3).

Whether on canvas or cardboard, Voronovsky’s subjects remained much the same. Birds abound in Voronovsky’s work, from his paintings of dizzying flight formations to his Styrofoam sculptures, which he arranged in groups in his home. His birds, like those that appear time and again in Ukrainian folk-art, belong alternatively to the animal kingdom and mythology, their purpose both decorative and narrative. At every point in his life – even in the harshest conditions of Nazi internment – Voronovsky would have been able to admire birds and their freedom as they flew beyond the reach of human cruelty, traveling great distances in elegant formations without losing their flock. By deploying a rich panoply of birds in his art, Voronovsky intertwines observed nature with artistic traditions, reaching toward a higher significance: a tribute to survival through migration.

The trains, buses and ships found throughout Voronovsky’s work are more prosaic indexes of the artist’s multiple (often mechanized) migrations, forced and voluntary, across an increasingly interconnected world. As the so-called ‘Breadbasket of Europe’, Ukraine possessed a sprawling rail network that connected its rich grain-producing regions with urban trading centres and ports, simultaneously ferrying passengers between their city dwellings and country homes (*dacha*), a pilgrimage among the early-20th century leisure classes that Voronovsky doubtless took part in (see Figure 3). Employed as a mapmaker, Voronovsky was likely a frequent rider, and the disparate landscapes that appear in his art – from the wooden churches of the Carpathians to the resort towns of Crimea – suggest a well-traveled professional.

Voronovsky continued to rely on these modes of transportation after the war. The Displaced Persons camps Voronovsky found himself in allowed for a much more fluid existence than did the resettlement and labour camps he lived in during the Nazi occupation. The camps were also rich with cultural and political life. During this period, Voronovsky played with the Musical Wanderers, a Ukrainian folk music group that toured the Displaced Persons camps throughout the 1940s. Groups like these would have contributed to a sense of cultural belonging among Ukrainians who had long lived under foreign powers and found themselves once again without a self-governed homeland (Antons, 2014). Voronovsky’s many paintings of folk-dance performances may hark back to troupes he accompanied and saw perform during this time.

**Figure 4.**
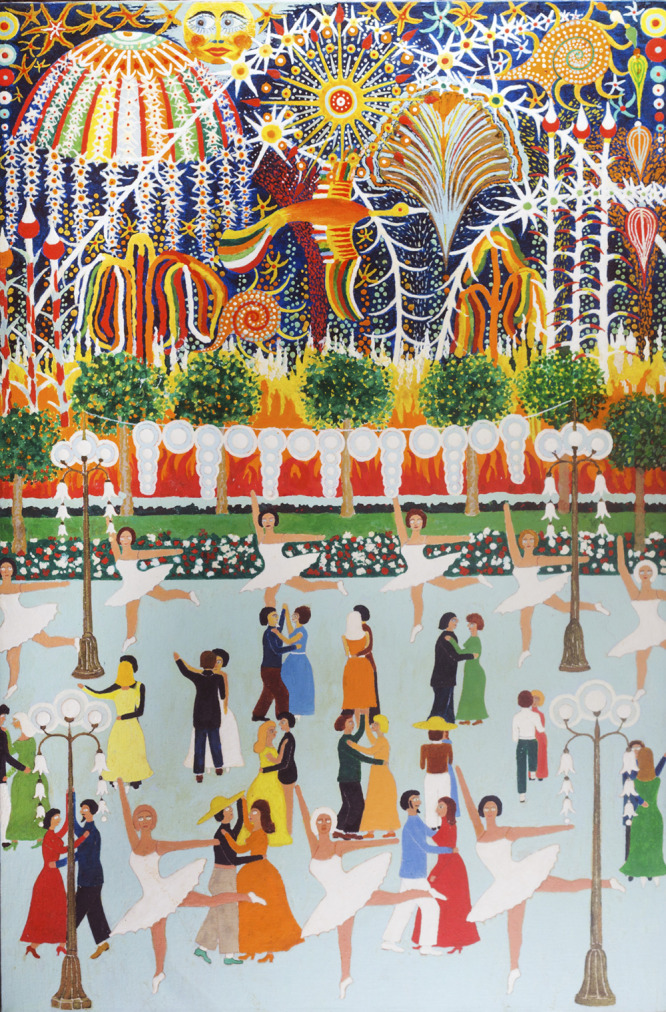
*Untitled (Dancers and Fireworks)*, 1978–1982, paint on canvas, promised gift of the Monroe Family Collection.

Of a very different tenor – more waltz than hopak – is the scene in Voronovsky’s *Untitled (Dancers and Fireworks)* (Figure 4). Filled with electric street lamps, contemporary costumes and well-choreographed ballerinas, the lower half of this painting is an ode to a modern and internationalized early 20th-century Ukraine (Hamm, 2010). By contrast, the top half is a chaotic mix of pure whimsy and colourful symbols from Ukrainian folk-art, which together double as a firework show. Soaring across the sky is a multicoloured firebird, a mythological creature of Slavic folklore symbolizing rebirth and happiness. By combining these disparate elements – real and imagined, modern and folk – Voronovsky achieves a stylistic and cultural synthesis that animates much of his work.

Paintings like *Untitled (Dancers and Fireworks)* also reveal an artist who straddled multiple – seemingly contradictory – worlds, as comfortable in a cosmopolitan environment as he was fluent in folk custom, adept at painting on both canvas and cardboard. Voronovsky’s *memoryscapes* spring from this matrix of styles, influences and experiences as childhood memories of a distant and largely decimated past find meaning in the modernity and materiality of the present, an act of creative resistance in the face of loss that makes his work both compelling as historical document and eternally relevant for its humanity. For a new generation of Ukrainian artists seeking haven from renewed conflict, the possibilities for healing found in George Voronovsky’s art are a reminder of what it means to create in the face of annihilation.
